# The past, present and future of neuroscience data sharing: a perspective on the state of practices and infrastructure for FAIR

**DOI:** 10.3389/fninf.2023.1276407

**Published:** 2024-01-05

**Authors:** Maryann E. Martone

**Affiliations:** ^1^Department of Neurosciences, University of California, San Diego, CA, United States; ^2^San Francisco Veterans Administration Hospital, San Francisco, CA, United States

**Keywords:** data sharing, neuroinformatics, data bases, FAIR (findable accessible interoperable and reusable) principles, data management, incf

## Abstract

Neuroscience has made significant strides over the past decade in moving from a largely closed science characterized by anemic data sharing, to a largely open science where the amount of publicly available neuroscience data has increased dramatically. While this increase is driven in significant part by large prospective data sharing studies, we are starting to see increased sharing in the long tail of neuroscience data, driven no doubt by journal requirements and funder mandates. Concomitant with this shift to open is the increasing support of the FAIR data principles by neuroscience practices and infrastructure. FAIR is particularly critical for neuroscience with its multiplicity of data types, scales and model systems and the infrastructure that serves them. As envisioned from the early days of neuroinformatics, neuroscience is currently served by a globally distributed ecosystem of neuroscience-centric data repositories, largely specialized around data types. To make neuroscience data findable, accessible, interoperable, and reusable requires the coordination across different stakeholders, including the researchers who produce the data, data repositories who make it available, the aggregators and indexers who field search engines across the data, and community organizations who help to coordinate efforts and develop the community standards critical to FAIR. The International Neuroinformatics Coordinating Facility has led efforts to move neuroscience toward FAIR, fielding several resources to help researchers and repositories achieve FAIR. In this perspective, I provide an overview of the components and practices required to achieve FAIR in neuroscience and provide thoughts on the past, present and future of FAIR infrastructure for neuroscience, from the laboratory to the search engine.

## Introduction

The transformation of neuroscience from a closed to an open science, where the entirety of research products like data and code produced during a study are routinely made available, has accelerated in recent years. Data sharing requires that the necessary human and technical infrastructure be in place to make these data broadly available. The first Human Brain Project, funded by the US National Institute of Mental Health in the 1990s, launched some of the first efforts to “database the brain,” envisioning a “paradigm shift in which primary data are openly shared with the worldwide neuroscience community” (Koslow, 2000). Despite this early optimism, neuroscience had a rocky history with open data sharing. Unlike the genomics and structural biology communities where the mechanisms and value of sharing primary sequence and structural data were agreed upon fairly early, the how and why of sharing the more diverse and complex data types of neuroscience was met with early resistance (Whose Scans Are They, Anyway?, 2000). In these early days, before the spotlight was shown on reproducibility problems facing neuroscience (Ioannidis, 2007; Button et al., 2013) and before “big data” became a buzzword in neuroscience and across biomedicine, there were few motivations or incentives for researchers to share their data openly. Like other areas of biomedicine (Nelson, 2009), neuroscience archives were largely underpopulated relative to the amount of data generated in Table 1 (Ferguson et al., 2014).

**Table 1:** State of population of selected data repositories 2014 vs. 2023.

Resource name	Country / region	Type of data	Date started	Data elements 2014	Update to resource (Feb 2023)	Data elements 2023	Datasets added since 2014	Provenance
NDAR	USA	Demographics, imaging, genetic, phenotypic	2009 (oldest news archives)	>108,000 subjects (from 157 labs)	Now NDA; no longer restricted to autism	–	–	Not comparable as new data types were added
NeuroMor pho.Org	USA	digitally reconstructed neurons	2006	11,335 (reconstructio ns from 1,339 publications)	Still in existence under same stewardship	298,387 reconstructions 2,103 publications	287,052 reconstructions 764 publications	https://neuromorpho.org/LS_availability.jsp Feb 25 2023
Cell Centered Database/ CIL-Cell Image Library	USA	images, videos, and animations of cell	2002 CCDB/2010 CIL	10,360 image datasets	Still in existence under same stewardship	13,990	3,630	http://www.cellimagelibrary.org/images?k=&simple_search=Search copied number of results Feb 25, 2023
FigShare	International	Various	–	> 8,000 datasets (query: neuroscience)	Still in existence under same stewardship	182,542	174,542	query: neuroscience with dataset filter Feb 252,023
ModelDB	USA	computational neuroscience models	1996	875 available datasets	Same stewardship; transition of leadership	1787	912	https://tinyurl.com/37z5p88f Feb 252,023
Open Source Brain	United Kingdom	Models	2014	47 available datasets	Still in existence under same stewardship	99	52	https://www.opensourcebrain.org/projects
CRCNS	USA	computational neuroscience	2008	38 available datasets	Under same stewardship; not clear if still active	140	102	documented through NIF; Feb 2023
XNAT Central	USA	Neuroimaging	2010	34 available datasets	Will be decommissioned in Oct 2023	510	300	https://central.xnat.org/ project number on home page; accessed Feb 252,023
1,000 Functional Connecto mes Project/IN DI	International (USA, China, Germany, Spain)	fMRI, DTI, MPRAGE, psychological assessements, behavioral phenotype, demographic	2009	28 datasets	Under same stewardship; also 1,000 Functional Connectomes INDI	33	5	
OpenfMRI	USA	fMRI	2012	24 datasets	Under same stewardship; changed name to Open Neuro	805	781	https://openneuro.org/ Feb 26 2023
BIRN	USA	Imaging, histology	–	21 datasets	No longer in service		–	
LONI Image Data Archive	USA	Imaging	–	18 (atlas), 9 databases	Under same stewardship; changed location; hard to compare as atlases and databases are not provided	144	135	https://ida.loni.usc.edu/login.jsp
BrainLiner	Japan	ECoG, EEG, fMRI, MEG, Microelect rode, NIRS, Optical Imaging, PET, Other	2011	10 available datasets	Platform there but does not look like it has been updated recently	23	13	http://brainliner.jp/search/showall/1
Open Connecto me Project	USA	Serial electron Microscopy	2011	9 available datasets	Now NeuroData	24	15	https://neurodata.io/project/ocp/ Manually counted Feb 252,023
CARMEN	United Kingdom	neurophysiology	2006	–	No longer in service according to NIF	–	–	
FITBIR	USA	Common data elements	2011	–	Same stewardship	–	–	
INCF Dataspace	International	Various	2012	–	No longer in service	–	–	
UCSF DataShare	USA	biomedical including neuroimaging, MRI, cognitive impairment, dementia, aging	2011	18 datasets	No longer in service	–	–	

Update of Supplementary Table 1 from Ferguson et al. (2014): A sample of Neuroscience centered data repositories available to the community. Only data repositories that accept outside data are included in the update. This table provided the number of data elements (usually equivalent to datasets) in each repository in 2014 (Data elements 2014). We include an update on the status of the resource (Update to Resource Feb 2023 column), the number of data elements found in Feb 2023 (Data elements 2023), the total number added since 2014 (Datasets added since 2014), and how these numbers were derived if the repository did not provide the number of datasets directly. Data repositories that are no longer in service are colored in light orange.

Neuroscience started to put its first big stake in the ground for open data sharing with the commissioning of large prospective data sharing efforts where large, comprehensive data sets were collected by large teams of scientists with the goal of making them openly available. Some of early efforts include the Alzheimer’s Disease Neuroimaging Initiative (ADNI; Weiner et al., 2010) launched in 2004 and Allen Brain Atlas launched in 2005, followed by large consortia such as the Human Connectome Project (2011) and the Big Brain (2013; Amunts et al., 2013) among many others. The large national and international brain projects launched in the second decade of the 21st century articulated a strong commitment to the open sharing of data and tools. The European Human Brain Project (HBP) was launched in 2013, followed by the US Brain Research through Advancing Innovative Neurotechnologies (BRAIN) Initiative (2014), the Korean Brain Initiative (2016), Canadian Brain Research Strategy (2017), Japan BRAIN/Minds (2018), and the China (2021) and Australian Brain Projects (International Brain Initiative, 2020; Quaglio et al., 2021). These projects have provided a significant infusion of resources to develop the next generation infrastructures necessary to house the sizes and complexity of data developed through new imaging, genomic, and physiological techniques.

An updated analysis of the repositories listed in Ferguson et al. (2014) provides some data on the current state of data sharing. Table 1 shows that data sharing has increased overall, but it is uneven, with explosive growth in some repositories, e.g., NeuroMorpho.org and FigShare, and more modest growth in others. But with the release of the data sharing mandates by funding agencies around the globe (Funders’ Policies, 2015; Eke et al., 2022), neuroscience-whether practiced by large consortia or individual labs-is now expected to be “open by default and open by design” (National Academies of Sciences, Engineering, and Medicine, 2018). So the question is no longer whether neuroscience as a whole will share data, it is how effectively? We are seeing some real success stories emerging in neuroscience from the reuse of data, e.g., (Torres-Espín et al., 2021; Almeida et al., 2022) and the ability for multiple groups to analyze the same datasets are providing new insights into notions of reproducibility and robustness (Botvinik-Nezer et al., 2020), but public data are still often difficult to find and use. Effective data sharing, that is, data sharing that views data as a public product of research meant to be reused, referenced, and respected requires the infrastructure, skills, tools, and willingness on the part of the neuroscience community to value data as a research product (Martone and Nakamura, 2022).

Effective data sharing starts with the FAIR data principles (Wilkinson et al., 2016) which grew out of frustrations experienced when trying to use open data on the web in the early days of sharing data. Through the Neuroscience Information Framework (NIF), started in 2008 (Gardner et al., 2008), we were tasked with cataloging all the neuroscience-relevant digital products that were being created (Cachat et al., 2012). NIF was also tasked with developing a strategy to query across the dozens of neuroscience data-and knowledge bases and the 100’s of biomedical databases with neuroscience-relevant information that were coming on-line. In these early days of on-line databases, the problems with accessing the data were legion: broken links, insufficient metadata, non-standardized vocabularies and nomenclature, non-actionable data formats, cryptic variables, and proprietary formats to name a few.

FAIR states the minimum set of requirements for digital data for it to be useful: data should be findable, accessible, interoperable, and reusable. FAIR then lays out a set of practices that would make it more likely that data will meet these requirements. The FAIR data principles were formulated in a workshop in Leiden in 2014 (Wilkinson et al., 2016), and were first released through FORCE11, the Future of Research Communications and e-Scholarship. The paper came out 2 years later in 2016. When our group participated in the 2017 kick off meeting for the BRAIN Initiative Cell Census Network (BICCN), a large consortium designed to use multimodal data techniques to determine the major cell types in the brain, few hands were raised when we asked how many people had heard of FAIR. Fortunately, FAIR eventually made its way to neuroscience and found a natural home in the International Neuroinformatics Coordinating Facility (INCF.org), an international organization devoted to developing standards and coordinating infrastructures for neuroscience. INCF incorporated FAIR into its mission statement and has served as a coordinating center for introducing neuroscience to FAIR through its role as a standards organization for neuroscience, its training programs, and other resources (Abrams et al., 2021).

## The FAIR partnership

**Figure 1:**
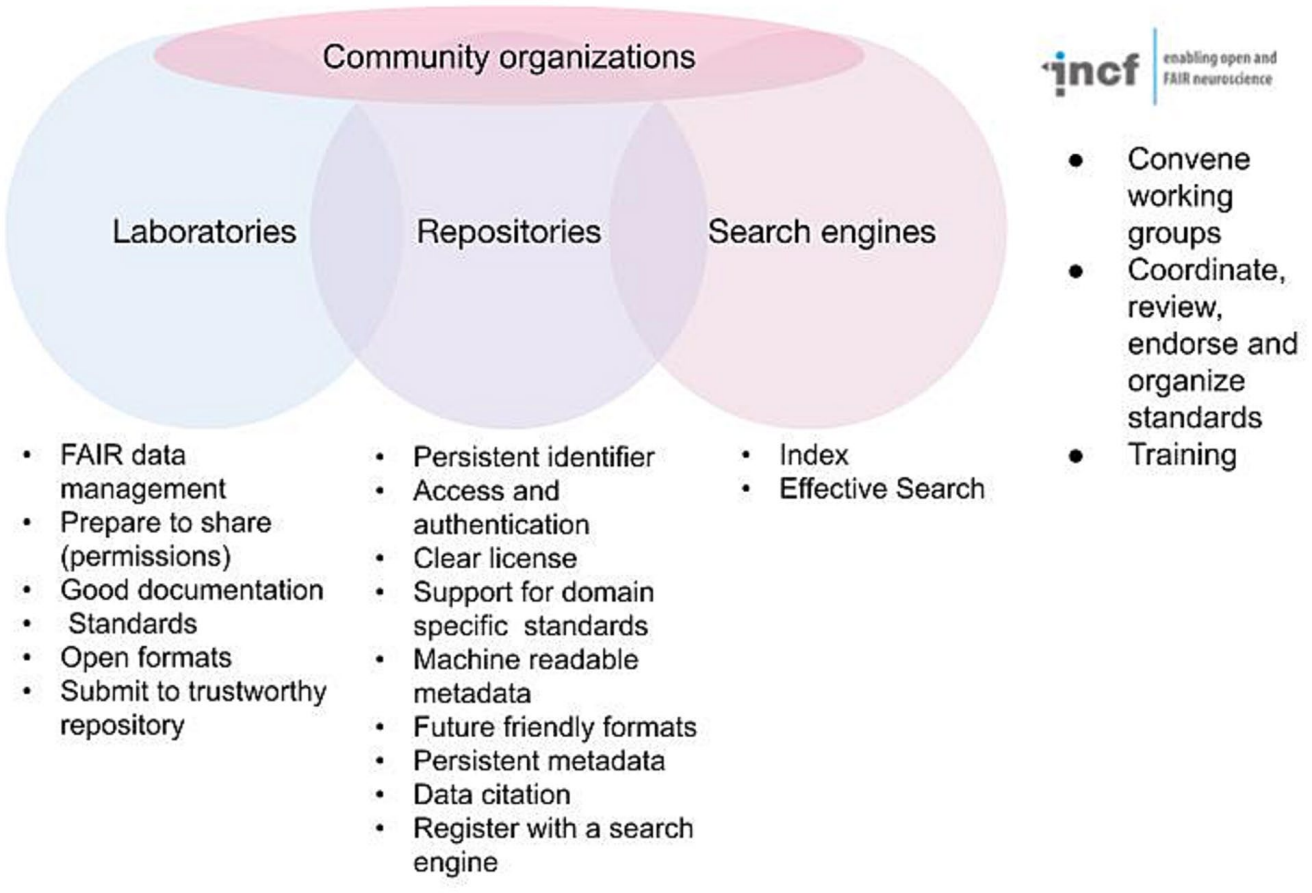
Major stakeholders involved in defining and implementing FAIR. Some of the major requirements for achieving FAIR are listed under each stakeholder group. The INCF is given as an example of a community organization supporting FAIR for neuroscience.

The FAIR acronym itself is now likely better known among practicing neuroscientists, as funders and journals have started to support FAIR in their data sharing policies; but the details of FAIR as elaborated in the detailed recommendations are fairly arcane. Anyone outside the field of informatics is likely to look at these and scratch their head. Persistent identifiers? Knowledge representation languages? A plurality of relevant attributes? Thus, while the practicing neuroscientist may understand what FAIR stands for, they are often at a loss to explain exactly how to achieve it. In reality, no one can create fully FAIR data alone; it requires the interplay of data acquisition and documentation practices, infrastructure, informatics, and community consensus. FAIR is therefore best thought of as a partnership between investigators, data repositories, data aggregators and community organizations (Figure 1). Navigating the landscape of FAIR data sharing and neuroscience infrastructure requires understanding the roles, responsibilities, and interfaces between each of these stakeholder groups. In the following I discuss the different components and some of the tasks required for FAIR and provide information and resources to help navigate the different components required for fully FAIR neuroscience.

### Laboratories

#### FAIR data management

In the US National Academies of Science, Engineering and Medicine workshop on “Changing the Culture on Data Management and Sharing (Martone and Nakamura, 2022), one of the main takeaways was that the focus of data sharing efforts should not be targeted toward the individual investigator, but the laboratory. As one participant noted: “If you can share data with people in your lab, you are much more likely to have something worthwhile to share outside the lab.” FAIR data management is therefore an intentional lab-wide strategy that ensures that data can be shared with lab mates, the PIs, and other colleagues, your future self and eventually with the broader scientific community. Across all stages of the data lifecycle, the management strategy puts in place processes so that data can be found, accessed, combined when necessary, and reused. By paying attention to FAIR in the laboratory throughout the life cycle, benefits start to accrue to the data creator, the laboratory, PI, and collaborators well before data flows out to the wider scientific community (Bush et al., 2022; Dempsey et al., 2022).

Examples of lab management practices built on the FAIR principles are given in Table 2.

**Table 2:** Some FAIR laboratory data management practices.

FAIR goal	Principle	FAIR practices	Reference
Findable	Unique identifiers	1. Create identifiers that are globally unique within the lab for all key entities in the lab, e.g., subjects, experiments, reagents, via the creation of a central registry or use of an existing system, e.g., RRIDs for reagents and tools. Globally unique = no two objects have the same ID, no ID may be reused.	Fouad et al. (2023)
	Rich metadata	Each identifier in the registry is accompanied by rich metadata that provides key details, e.g., for experiments: dates, experimenter, description, collaborators, techniques etc.; for subjects: species/strain, age, weight, etc.	Fouad et al. (2023)
		Use unique identifier for file names, folder names, to label physical objects like slides or slide boxes, so that all entities associated with the lab can be tied unambiguously to metadata	
Accessible	Authentication and authorization	Create a centralized, accessible store for data and code under a lab-wide account for lab data to ensure that files are not scattered around multiple systems or accessible only via personal accounts that may not be available after someone has left the lab.	
Interoperable	FAIR vocabularies	Move away from idiosyncratic naming of variables and annotations towards standards like Common Data Elements and the use of community-based ontologies, atlases, and controlled vocabularies. Consistent lab, wide terminology ensures that lab members can understand what the data are about, and aids in search across and combining files.	Bush et al. (2022)
		Consider creating a lab-wide data dictionary where all variables used across experiments are clearly defined	Bush et al. (2022); Fouad et al., 2023
Reusable	Documentation	Create a “Read me” file for each dataset where notes can be captured and helpful information provided for reuse of the data	
	Community Standards	All files should be collected and stored in well supported open formats ideally to ensure long term availability.	
		Adopt community standards within the lab where possible; a good place to identify relevant standards is to look at repositories where the data may end up. Specialized repositories usually have a list of required or recommended standards. Some repositories are providing help with developing a data management and sharing plan for grant proposals, e.g., INCF, SPARC and ODC-SCI/TBI.	Bush et al. (2022); FAIRsharing.org, INCF Standards Portfolio
	Provenance	Datasets should be clearly versioned and differences between them documented. Depending on the system used for storing data, formal support for versioning may be available, e.g., Google Docs, but if not, implement a file naming convention so that versions can be tracked	
		Always keep a version of record that can be reverted to if necessary. Often when one is working with data, different versions are created rapidly and it is easy to lose track of which version is which. It is good practice to have stable versions that are easily retrievable so that there are stable points to which to return if provenance is lost.	
		Datasets should also be accompanied by detailed experimental protocols that describe how the data were acquired and computational workflows that detail the processing steps. Use of tools designed for this purpose, e.g., protocols.io, NeuroShapes (Neuroshapes, n.d.) and ReproNIM (Kennedy et al., 2019).	
	Licenses	Prepare to share: Make sure that how and when the data are to be shared is agreed upon with all collaborators early on. For clinical datasets, make sure that the consents are in place for open sharing of de-identified data.	

Examples of laboratory data management practices based on the FAIR principles.

We are starting to see neuroscience researchers sharing their experiences with developing and utilizing lab-centric data management systems. They range from tightly integrated digital infrastructures (Bush et al., 2022; Dempsey et al., 2022) to a set of practices that can be implemented using “off the shelf” components for an average neuroscience wet lab (Fouad et al., 2023).

#### Choosing a repository

One of the most important steps for a researcher in ensuring that their data is FAIR for the long term is to submit their data to a trustworthy repository that supports FAIR. The new NIH data sharing policy requires researchers to indicate where they will be sharing their data as part of the data management and sharing plan. As recommended in Table 2, knowing in what repository the data will be published allows the researcher to understand what standards are required so they can be built into the laboratory management workflow. With its growing ecosystem of specialized databases, researchers have a choice about where to publish their data.

**Figure 2:**
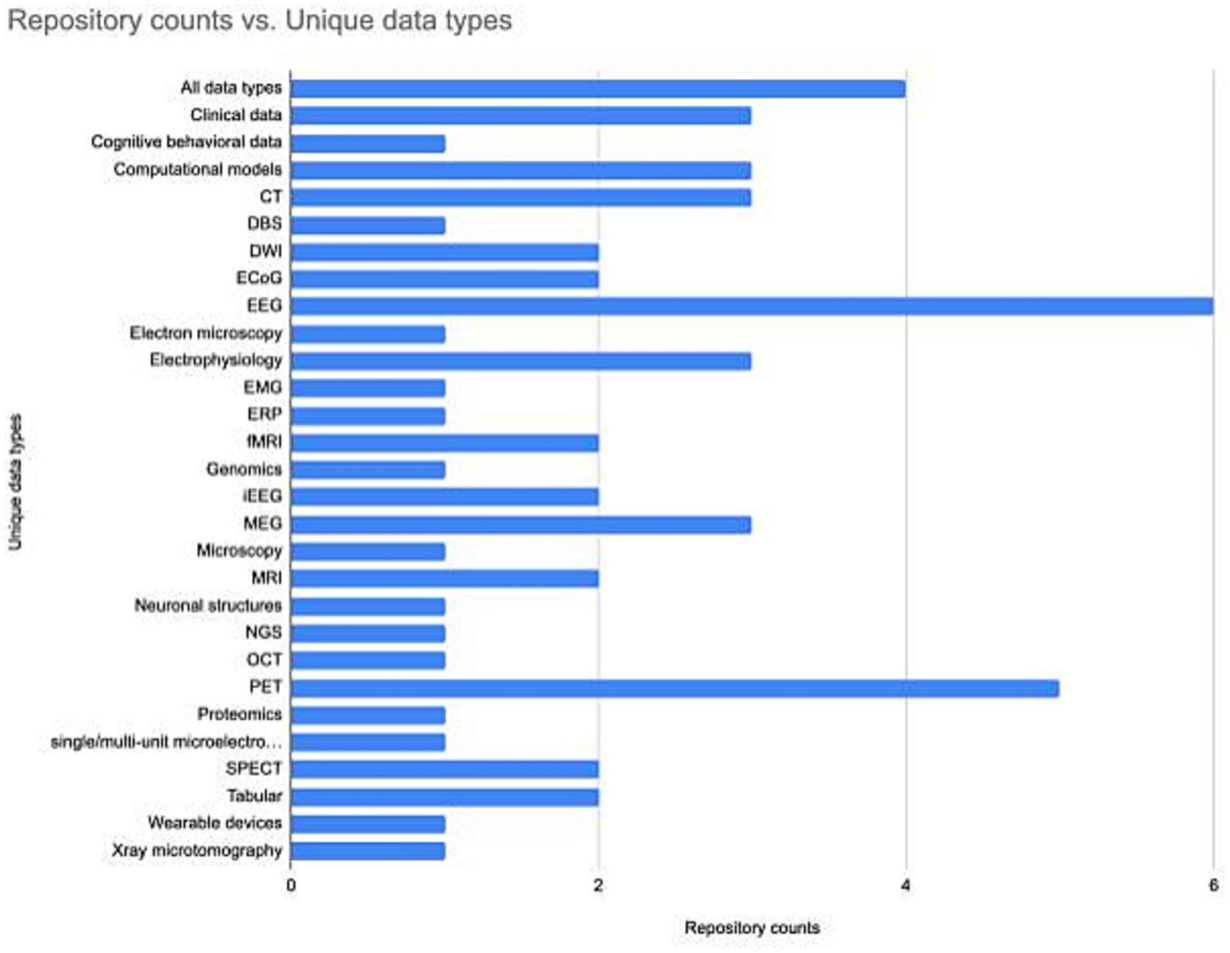
The number of neuroscience specialist repositories supporting different data types. The repository list and associated data types was assembled using information available through the INCF Infrastructure Portfolio and the SciCrunch Registry. The data underlying the figure is available at Zenodo, DOI: 10.5281/zenodo.8239845.

Understanding how the neuroscience repository landscape is organized may help in finding the right repository. Repositories are generally specialized by data type (Figure 2). However, repositories also exist that are specialized for a domain, e.g., the SPARC database accepts all data associated with the peripheral nervous system, or serve researchers within a particular region, e.g., CONP, or institution, e.g., BrainCode and the Donders Repository. Often, multiple repositories may be appropriate, in which case there are additional features that may make a given repository more or less attractive. These include tool support, curation services, support for data citation, choice of license, size of data allowed, help with data management plans (see Table 2) and possible costs (Murphy et al., 2021). A functioning neuroscience ecosystem also requires open neuroscience repositories that have few restrictions on data types, regions, or subdisciplines to ensure that all data has a home. The EU EBRAIN infrastructure is an example of such a repository, as it takes multiple types of data regardless of discipline or geographical location, although there may be issues with transferring certain types of data across international borders (Eke et al., 2022).

Supplementing the specialist repository landscape are the generalist repositories, data repositories that span scientific disciplines and data types (Assante et al., 2016). These repositories are often useful for publishing smaller supplemental datasets that are required for a publication (Stall et al., 2023). Specialist repositories generally provide more standards, tools and services for harmonizing and using data, and make it easier for researchers to find data of a particular type. To aid researchers in choosing an appropriate neuroscience data repository, the INCF has a searchable infrastructure catalog, where each repository is described according to the checklist developed by Sandström et al. (2022). Other repository finder tools include NITRC for neuroimaging related repositories, re3data, the catalog of open data repositories maintained by the National Library of Medicine, and the NIF listing of BRAIN Initiative Repositories.

### Repositories

#### The central role of community repositories

While the investigator takes the central role in acquiring data in a manner that supports FAIR, the community repository is arguably the central player in implementing the basic requirements for achieving FAIR for the long term (Figure 1). We are using the term “community repository” here to designate infrastructures that are designed to accept primary data contributed by outside researchers, rather than a single data set produced by a given project (e.g., the Allen Brain Atlas) or a knowledge base that aggregates information about a particular entity (e.g., CoCoMac).^1^ As shown in Figure 1, the repositories have critical responsibilities for ensuring that submitted data are made available according to the FAIR principles (Lin et al., 2020). These practices include issuing and maintaining persistent identifiers, tying those identifiers to rich metadata, providing access and any necessary access controls, enforcing or supporting annotation with FAIR vocabularies, enforcing or supporting community standards, supporting data versioning, providing links to other critical products like experimental protocols and code, and provisioning a clear data license for each data set. Repositories also have the critical role of ensuring that data is available for the long term.

From the earliest days of neuroinformatics, it was envisioned that neuroscience would likely best be served by a decentralized system of federated databases (Koslow, 2000). Due to the variety and complexity of neuroscience data, a single large repository like Genbank or the Protein Data Bank was likely not going to be feasible. The early investments in neuroinformatics by the US Human Brain Project and the success of the International Neuroinformatics Coordinating Facility in growing the field of neuroinformatics globally, led to the first generation of neuroscience databases. These databases were largely organized around data type, e.g., structural neuroimaging (XNAT), functional neuroimaging (fMRI Data Center; Open fMRI), neurophysiology (CARMEN; Neurodatabase.org,” GNode), EEG (open EEG, iEEG), neuronal morphology (NeuroMorpho), microscopic images (Cell Centered Database), neuromodeling (ModelDB). Some examples are shown in Table 1.

When the first generation of neuroscience databases were started, there were few standard practices for designing web-accessible databases. As documented by NIF, each database had a different mode of access, different data structure, and the use of standards was very limited. It was a time of tremendous technological fluidity, with standard features we take for granted today (e.g., RESTful web APIs) still being invented. The cloud did not exist, and attempts to build resources on the early version of a cloud-like system (“the grid”) met with considerable challenges (Grethe et al., 2005). With today’s emphasis on data sharing, increased attention is starting to be paid to these critical infrastructures and how they are constructed, operated, and evaluated (Nelson, 2022). Various recommendations on desired characteristics for data repositories have been issued by different groups (Sansone et al., 2020; Shearer, n.d.), including NIH (Selecting a Data Repository) and additional sets of principles, e.g., the TRUST principles (Lin et al., 2020) and principles for open infrastructures (Bilder et al., 2015) have been formulated to help further guide how these critical infrastructures should operate. The Elixir project, a large scale bioinformatics consortium in the EU, has developed a maturity model for evaluating the success of repositories which is designed to be used by funders to determine the criticality of various infrastructures (Bahim et al., 2020). The INCF Infrastructure working group recently issued a set of guidelines from a neuroscience perspective, that provide a mix of technical and “customer service” recommendations for operating repositories (Sandström et al., 2022). Although these various lists of desiderata do not overlap completely (Murphy et al., 2021), over time we will likely converge on a core set of functions and expectations for these critical infrastructures, balancing the often dual requirement for these infrastructures to serve as both publishing platforms and dynamic scientific gateways (Sandström et al., 2022).

INCF has served as an important conduit by which the FAIR principles have permeated the construction of neuroscience data repositories and gateways. Investigators who have been active in INCF through governance, committees and working groups are involved with several of the next generation neuroscience infrastructures including EBrains, CONP, SPARC, DANDI, Open Neuro, and BRAIN/Minds. Table 3 lists and compares some of the key ways that these infrastructures implement FAIR and “FAIR-adjacent” practices. Following consistent design principles that support FAIR provides a level of common functionality and services that make it easier to work across these databases for an individual user or an automated agent. The more similar FAIR practices are across repositories, the more likely it is that the repositories themselves are interoperable.

**Table 3:** FAIR practices across data repositories.

Principle	Function	EBRAINS	SPARC		DANDI	CONP Portal	OpenNeuro
F1. Globally unique identifier	Basic core	DOI	DOI		DOI	ARK, DOI	DOI
F2. Rich metadata		Y	DataCite		Y	DATS	Y
A1. Retrievable by identifier		Y	Y		Y	Y	Y
A1.1 Free, open, universal retrieval protocol	Enhanced access	Y	Y		Y	Y	Y
F4. Registered in a searchable resource		KS, GDS	KS, GDS		KS, GDS	KS	KS, GDS
A1.2: Authentication and authorization		Y	Y		Y	Y	Y
R1.1: Clear data usage license		Y	CC-BY		CC-BY, CC0	Y	CC0
R1.3: Community standards	Use of standards	Multiple	SDS, MIS		NWB, BIDS	Y*	BIDS
F3: Metadata contains identifier		Y	Y		Y	Y	Y
I1: Formal knowledge representation language		Y	Y		N	Y	
R1: Plurality of relevant attributes	Rich(er) metadata	OpenMinds	OpenMinds, MIS		NWB	DATS	Y
I2: FAIR vocabularies		Y	Y		Y	Y	N
I3: Qualified references to other metadata		Y	Y		Y	Y	Y
R1.2: Provenance	Provenance and context		Exp Protocol			Y	N
A2: Metadata persistence			Y		Y		
Landing page	Additional features	Y	Y		Y	Y	Y
CCFs		Y	Y*		N	N	N
Data citation		Y	Y		Y	Y	Y
Curation		Y	Y		N	Y	N

Comparison of FAIR features across five large brain repositories where the principal investigators have been active through the INCF. The principles are organized according to the functions they support based on an organization proposed by Hodson et al. (2018). Highlighted in purple are additional features that are relevant for FAIR although they are not mentioned explicitly in the FAIR principles, e.g., the use of landing pages and support for data citation. KS, INCF Knowledge Space; GDS, Google Dataset Search; DOI, Digital object identifier; NWB, NeuroData Without Borders; BIDS, Brain Imaging Data Structure; DATS, Data tag suite.

#### Standards: role of repositories

A significant and positive change that is accelerating progress toward FAIR is the emergence of a set of robust standards for neuroscience data types that are starting to gain adoption. The INCF was created to help with this process of standardization and produced some early successes, e.g., the Waxholm space for registration of mouse and rat brain data (Hawrylycz et al., 2011; Papp et al., 2014), the Neuroimaging Data Model (Keator et al., 2013) the Brain Imaging Data Structure (Gorgolewski et al., 2016) were produced with support from INCF. Over the last few years, a set of standards has emerged for major neuroscience data types that can accommodate the increased size and complexity of neuroscience data through additional investments by funders and the efforts of the large brain projects, e.g., NWB, 3D-MMS (Ropelewski et al., 2022). Repositories serve as important stakeholders in ensuring that standards are followed by supporting or requiring them for data submission (Figure 2). Data uploaded to OpenNeuro, for example, must be validated against BIDS before it is accepted. The INCF has implemented an open community review and endorsement process to help improve the quality, usability, interoperability and awareness of these standards (Abrams et al., 2021). They have made available a searchable Standards and Best Practices Portfolio^2^ where researchers can learn about each standard and how it can be used. FAIRsharing.org more broadly aggregates standards from across biomedicine and makes them available through a searchable catalog.

**Figure 3:**
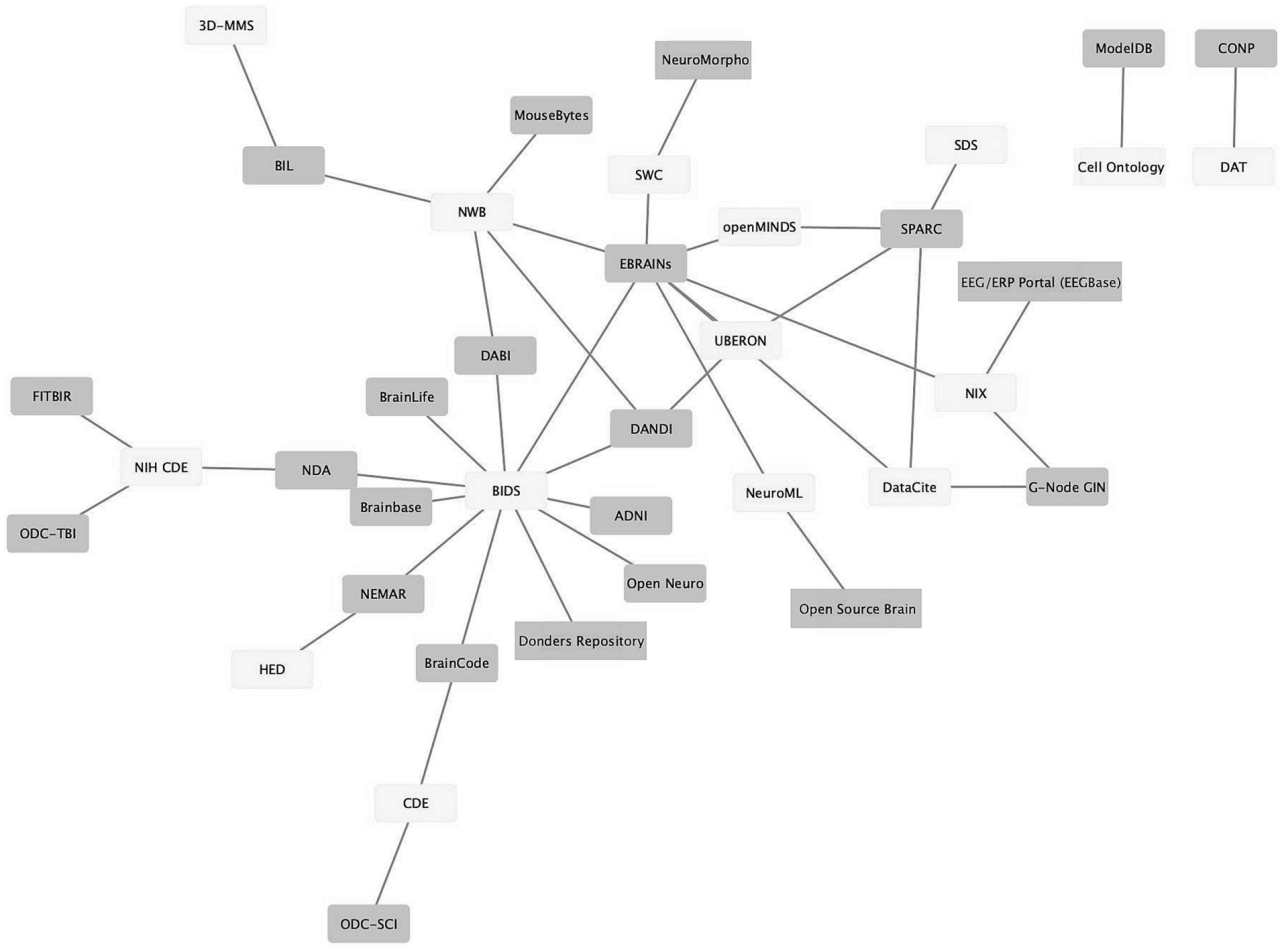
Ecosystem of neuroscience resources emerging around standards. Network graph of neuroscience data repositories and gateways (purple) and some of the standards they support (yellow). The graph shows repositories/gateways connected via the use of a common standard. A description of how standards were determined is given in the text.

As neuroscience standards become more mature, better supported, and more widely used, they provide the seeds for knitting the landscape of neuroscience data repositories into a true data ecosystem, where (meta)data can flow from the laboratory to repositories and from repositories to computational tools and back again. Figure 3 shows a graph illustrating the connections between standards (light gray) and infrastructures that support them (dark gray). The data was assembled from the INCF Infrastructure Catalog, FAIRsharing, the SciCrunch Registry (Subash et al., 2023) and examination of repository websites. As shown in Figure 3, multiple repositories and infrastructures are connected via these standards. For example, the Brain Imaging Data Structure (BIDS; Gorgolewski et al., 2016) links 10 different repositories and computational platforms. The success of BIDS has led to extensions of BIDS for other modalities through a formal governance process (Governance, n.d.). The adoption of these BIDS-based standards starts to create a degree of interoperability across data types.

As tool support grows, standards are also making their way into the laboratory. BIDS, for example, has been estimated to have been used to organize over 100,000 datasets containing millions of images, indicating significant uptake by the research community (Poldrack et al., 2023). In a recent paper that outlined a neuroimaging center’s implementation of BIDS, Bush et al. (2022) stated: “*Learning the BIDS specification, implementing software pipelines to map the data, and validating that the resultant mappings met the BIDS standard consumed many months of effort across multiple imaging center team members… The benefits of mapping our data to BIDS, however, far exceed the costs.”* (Bush et al., 2022). These benefits included access to BID-APPS, a set of containerized analysis tools and pipelines that run on validated BIDS data, as well as improved code sharing within the lab and with colleagues, as well as a reduced barrier to publishing the data in OpenNeuro. Similarly, the electrophysiology standard, NWB, has made inroads in tackling one of the most challenging data types in neuroscience, evidenced by uptake in laboratories (Rübel et al., 2022) and support by platforms such as DANDI and EBRAINs.

#### Standards: use of FAIR vocabularies and common coordinate frameworks

Interoperability across neuroscience data has always been hampered by the multiplicity of nomenclatures and parcellation schemes from brain regions and nerve cells (Martone et al., 2004). Although slow, progress has been made. Some repositories are starting to map generic neuroanatomical structures to community ontologies like UBERON (Mungall et al., 2012). Mapping data to a common coordinate framework (CCF) allows more precise localization independent of labels applied to them (Hawrylycz et al., 2023). Encouraging signs are emerging, as CCFs for multiple species are in use or in development for the major species across the international brain projects. For example, both the BICCN/BICAN and EBrains are utilizing the Allen Institute Common Coordinate Framework v3 for mouse (Hawrylycz et al., 2009, 2023). To help manage the different versions and components that go into these atlas-based environments, a new standard for describing and versioning brain atlases was recently proposed (Kleven et al., 2023).

Standardized nomenclature for cellular taxonomies and transcriptionally defined cell types are also emerging from projects like the BICCN/BICAN to help deal with the plethora of new cell types that are emerging from new transcriptomics-based approaches (Miller et al., 2020; Tan et al., 2023). Over the years, there have been proposals for naming neurons that can bridge the multiplicity of phenotypes generated by multiple experimental techniques (Hamilton et al., 2012; Shepherd et al., 2019; Gillespie et al., 2022). However, these approaches have had difficulty in handling the complex expression patterns coming out of transcriptomics. The BICCN/BICAN recently developed the Brain Standards Data Ontology, providing a model for providing data-driven definitions of taxonomic classes (Hawrylycz et al., 2023; Tan et al., 2023). BICCN has recently introduced Cell Cards to provide a tool for exploring the BICCN taxonomic cell types for human, marmoset, and mouse primary motor cortex, including linking them to primary data sets (Hawrylycz et al., 2023). As new technologies are allowing us to derive wider scale, more complete representations of the molecular, morphological, physiological, and connectional phenotypes of neurons than was possible in the past, it is time for the global neuroscience community to come together around a common nomenclature for naming populations of cells that will aid in comparison across studies.

Services for accessing ontologies and building them into annotation and metadata pipelines have improved significantly over the past decade, with tools such as BioPortal^3^ and the Ontology Look Up Service^4^ providing programmatic access to community ontologies. Nevertheless, neuroscience is still a cutting edge science where many new terms are needed, particularly for annotating experimental data. For this reason, NIF and INCF had developed the NeuroLex Wiki (Larson and Martone, 2013) that lowered the barrier for creating new ontology terms. When the semantic wiki technology underlying NeuroLex was no longer available, the approach and content were ported to the Interlex on-line vocabulary management system by NIF (Surles-Zeigler et al., 2021). Interlex mints a unique identifier for each term (URI) when it is entered and allows the addition of basic metadata for each term, e.g., definition, synonyms. It also provides basic knowledge engineering functions, e.g., parent–child and other relationships, annotations. Interlex also provides various review and curation functions. These specialized terms can be used as controlled vocabularies or further engineered into ontologies as needed. Surles-Zeigler et al. (2021) provide a description of how Interlex is being used to enhance anatomical annotation of SPARC data, models and knowledge base, allowing new anatomical terms to be minted, curated, linked to existing ontologies and contributed as necessary to augment community ontologies.

### On the sustainability of neuroscience data repositories

As most neuroscience infrastructure is researcher-led and grant-supported, questions often arise about long-term sustainability when choosing a repository, or indeed, any infrastructure. Sustainability of individual resources remains a challenge, not just for neuroscience but for all research-led infrastructures that rely on grant funding for their operation. Of the data repositories listed in Table 1 taken from Ferguson et al. (2014), 4/18 are no longer in service and 3/18 are moribund (i.e., not taking data). Three were rebranded and expanded their scope, and one merged with another database. The good news is that the majority of this first generation of neuroscience databases are still in existence, indicating a degree of stability. We can also see movement in the ecosystem, with databases merging with others, or moving across institutions indicating a degree of dynamism that keeps the ecosystem healthy. Looking at a larger sample using the SciCrunch Registry (formerly the NIF Registry; Ozyurt et al., 2016) out of a total of 563 neuroscience data resources (including data repositories, databases, data sets, atlases and knowledge bases), 71 appear to be out of service (~13%). These numbers compare favorably to a study done on the longevity of bioinformatics biological databases founded in the late 20th century, 63% of which were defunct by 2015 (Attwood et al., 2015). In 2016 NIF began to track the usage of these neuroscience resources within the scientific literature (Ozyurt et al., 2016), revealing interesting patterns including the creation of thousands of data repositories across biomedicine. A recent analysis showed that only a handful of these repositories are actively used, with many of the neuroscience repositories referenced here among them, suggesting that neuroscience is coalescing around a set of core resources (Piekniewska et al., 2023). Thus, while sustainability is always a concern, neuroscience repositories have generally been good stewards of their data, utilizing a variety of strategies to keep data safe and accessible.

As neuroscience data and repositories start to align around the FAIR principles, the ecosystem should become more robust as it will make it easier for other repositories to absorb data if a repository loses its funding. Merging of similar resources also makes the ecosystem more efficient. The ‘professionalization” of scientific data repositories also means that researchers are taking their role as an archive more seriously. The INCF recommendations for neuroscience infrastructure include that repositories should have an exit plan and they should clearly state their persistence policy (Sandström et al., 2022). For example, some repositories are partnering with institutional libraries or other resources to ensure that data remain available, even if funding is lost (e.g., EBRAINS). Another promising development is the repurposing of infrastructure components. Rather than building a separate data repository, two computational and analytic platforms, Brainlife and NEMAR, utilize Open Neuro as their data platform, even as they field their own portals with their own branding. The ODC-SCI and ODC-TBI share the same infrastructure (SciCrunch; Surles-Zeigler et al., 2021), but each have their own separate community portal where they can access data and establish their own governance rules. The more that neuroscience infrastructure can be repurposed for new projects, the less funding needs to go to building and maintaining new infrastructures.

### Search engines

In tandem with the vision of a distributed system of databases laid out by the NIH HBP was the creation of a neuroscience portal where data could be accessed via a *“a smart ‘neuroscience browser’ instructed to look for a particular variable or set of variables and import the data back to the user’s computer”* (Koslow, 2000). For the distributed ecosystem to work effectively, users would have to be able to issue dynamic queries across these databases and be able to retrieve the necessary subsets of data. And, in fact, FAIR states that data should be registered with an appropriate index (F4). NIF set up one of the first searches across neuroscience databases by creating an index over the contents of distributed databases. At its height, NIF queried over 200 data sources across biomedicine comprising over 8 million data records (Cachat et al., 2012). NIF used the NIFSTD to help mediate across the different vocabularies and relationships that were needed to link across databases. NIF was able to align different databases covering the same content across a core set of variables, but did not have the resources to harmonize the content, especially given the lack of standards at that time. NIF was designed to allow researchers to understand what was in a given database by providing limited views of the data, but not to perform deep structured queries of the content. So you could use NIF to identify a database that had relevant data, but for more structured queries and to retrieve the complete data, users needed to visit the source database. The INCF Knowledge Space and currently performs a similar type of search over 16 major neuroscience databases (KnowledgeSpace, n.d.).

The more that repositories enforce consistent standards for metadata and data formats, the closer neuroscience gets toward achieving true federated search and retrieval across the entirety of the neuroscience repository ecosystem (Koslow, 2000). The Canadian Open Neuroscience Portal was recently launched that allows users to search across data hosted in multiple data repositories. It is currently deployed across 17 Canadian institutions and also integrates select specialist and generalist repositories. All the high level metadata is aligned to the DATS standard, developed by the NIH-funded BioCADDIE Big Data to Knowledge project (Alter et al., 2020), allowing for a unified dataset search. The portal implements some uniform functions that can be executed directly from the portal. Some data are available for download via DataLad and containerized workflows that work across these distributed data are available via Boutiques (Poline et al., 2023).

New tools are also becoming available that lower the barrier to making content available to search engines. For example, multiple neuroscience databases have marked up their content with schema.org so that their datasets are searchable through Google Dataset Search (Table 3). Neuroscience, like other domains, is building knowledge graphs that link neuroscience concepts to each other and to datasets to aid in search.EBrains, CONP and the SPARC projects are making their data available via a knowledge graph. CONP uses the Nexus knowledge graph developed by the Blue Brain Projects which provides a set of tools and resources for searching, linking and viewing data.^5^

### Community organizations

The FAIR data principles delegate a good amount of responsibility to individual communities to define what is FAIR for their domain. Community organizations play an important role as coordinators by serving as conveners to allow researchers to come to consensus about best practices and recommendations for their community. International neuroscience is currently supported by two community organizations, the INCF and the IBI. IBI is principally focused on coordination of the large international brain projects, focusing on data sharing among these projects, as well as issues such as data governance and ethics. INCF works across all neuroscience efforts, whether individual or team based, and focuses on standards, infrastructure coordination and training. Both organizations provide support for working groups that come together to tackle issues such as the development of international data governance (IBI), standards and best practices (INCF, IBI), training (INCF), and coordination of infrastructures (INCF, IBI). Any member of INCF can propose a working group and membership is open to the community, while IBI working groups are set by the Strategy Committee. The two organizations work together and with other organizations such as the IEEE Neuro Standards working group and the Global Brain Consortium.^6^ In this way, there is a level of coordination across these international organizations. Eke et al. (2022) raised the issue of whether neuroscience needs an umbrella organization modeled after the Global Alliance for Genomic Health, to more effectively address data reuse at the technical, ethical, sociological and political level.

## Is neuroscience FAIR yet?

Neuroscience has made tremendous progress over the first two decades of the 21st century in establishing the infrastructure, standards, expertise and tools for moving neuroscience significantly toward FAIR. It is now served by a set of robust international data repositories and scientific gateways specialized for neuroscience data, implementing the vision laid out in the dawn of neuroinformatics for a distributed ecosystem of repositories. The first inroads have been made in establishing FAIR practices and supporting infrastructure in the lab to manage data in a way that smooths the transition between private, semi-private, and public sharing. As best practices for FAIR are articulated, tested, and shared, we can expect that the quality of both the databases and the data will continue to improve.

A federated system allows neuroscience infrastructure to respond more rapidly to new data types and technologies as they are developed. While there are more resources to be sustained, there are also more resources from which to draw should a repository need to be decommissioned. We see from the last 20 years that there is movement in the repository landscape, with some resources ceasing operations, but others merging or changing ownership. As repositories start to align around sets of core features, both interoperability and flexibility will be increased, providing some measure of stability in an otherwise dynamic ecosystem.

While the distributed nature of neuroscience infrastructure brings many benefits, there are concomitant challenges it imposes on both those who submit their data and those that wish to use it. As the motivations and incentives for these two user groups can differ (Subash et al., 2023), balancing the efforts required to submit vs. reuse data will need to be a priority. Until these are addressed, neuroscience will not be considered a fully FAIR discipline:

- **Findable**: We still do not have an effective query system over the ecosystem of neuroscience data, that allows for aggregation relevant data distributed across multiple repositories. Important steps have been taken by IBI, INCF and CONP, but these efforts will need support if they are to be fully realized.
- **Accessible**: Users are increasingly acquiring multimodal datasets that may require deposition in multiple repositories. Currently, that requires a user to navigate multiple repositories, set up multiple accounts, entering the same metadata repeatedly and creating the necessary linkages across the different parts of the dataset (Subash et al., 2023). Some work is underway in the US BRAIN Initiative BICCN and BICAN projects to create a more unified workflow including a centralized registry, but such a service would be useful across all neuroscience. Many repositories are starting to implement login and authorization via ORCID, making it easier for users to work across multiple repositories.
- **Interoperable**: In a distributed system, interoperability is not just about the data but also about the infrastructures. Working across multiple repositories means working across multiple front ends, back ends and data access policies. As core sets of features are described for data repositories, neuroscience infrastructure may also start to converge on certain design patterns that make it easier for users to work across them. A term was introduced in an NIH Workshop on a FAIR Data Ecosystem for Generalist Repositories: coopetition (NIH workshop on the role of generalist repositories to enhance data discoverability and reuse: Workshop summary, 2012). Repositories can compete on certain features to encourage innovation, but there should be a set of features that are shared across repositories and work similarly.
- At the same time, competition among different data providers also can lead to a decrease in data interoperability, as repositories must compete for users. Thus, many repositories lower their requirements for standards compliance (Subash et al., 2023) recommending rather than requiring standards so as to lower the barrier of data submission. Instead of making compliance optional, neuroscience repositories should work on improving their customer service, providing both human and tool support to make it easier for researchers to comply with standards. SPARC has taken this approach, employing customer-oriented curators who assist researchers to comply with SPARC standards. SPARC also developed the SODA tool directed toward researchers with few computational skills to guide and support them in organizing and uploading their files according to the SPARC SDS (Bandrowski et al., 2021). In this way, the burden on the submitter is lessened, while data quality and standards compliance are not sacrificed.
- **Reusable**: Despite FAIR, most neuroscience data is still very difficult to use. Different projects have devoted different amounts of resources to curation of data and quality control. Generally curated data is of higher quality because it is more completely documented and some QC is performed (Gonçalves and Musen, 2019). Particularly with the push to make data AI/ML ready, funders should be prepared to support curation services for the near future, to ensure that high quality data are available. Such investments will likely not be needed forever; indeed, labs are at this moment experimenting with tools such as ChatGPT to help with query and harmonization. However, investments now in well curated data can help to accelerate training of these types of algorithms, while at the same time, making high quality data immediately available for discovery science.

Finally, usability is not simply a matter of technology or documentation. As Eke et al. (2022) and (Fothergill et al., 2019) have noted, the international nature of neuroscience infrastructure also means that issues of transferring data across national borders, i.e., international data governance, also must be addressed. Federation across distributed databases provides a model that can minimize data governance issues, as the data can remain in place, while compute is brought to the data (Poline et al., 2023).

The good news is that routine data sharing, if not exactly easy, is now at least possible across the sizes and complexities of neuroscience data. Islands of interoperation are starting to emerge among these different resources promoting federated search and shared computational platforms and services. Those of us who were involved from the beginning in attempts to “database the brain” cannot help but be impressed with how far neuroscience sharing and infrastructure has come, even as there is still quite a way to go. As the paradigm continues to shift toward open and effective data sharing in neuroscience, we will fulfill the early vision of neuroinformatics as a driver for *“..a new depth of understanding of how the nervous system works in both health and disease.”* (Koslow, 2000).

## Author contributions

MM: Writing – original draft, Writing – review & editing.

## Figures and Tables

**Table:** 

3D-MMS	Metadata for 3D microscopy standard
ADNI	Alzheimer’s Disease Neuroimaging Initiative
BICAN	BRAIN Initiative Cell Atlas Network
BICCN	BRAIN Initiative Cell Census Network
BIDS	Brain Imaging Data Structure
BIL	Brain Image Library
BRAIN Initiative	Brain Research through Advancing Innovative Neurotechnologies
CDE	Common data element
CONP	Canadian Open Neuroscience Platform
CT	Computed tomography
DANDI	Distributed Archives for Neurophysiology Data Integration
DATs	Data tag suite
DBS	Deep brain stimulation
DOI	Digital Object Identifier
ECOG	Electrocorticography
EEG	Electron encephalography
EMG	Electromyography
ERP	Event-related potential
fMRI	Functional magnetic resonance imaging
FORCE11	Future of Research Communications and e-Scholarship
HBP	Human Brain Project
HED	Hierarchical event descriptor
IBI	International Brain Initiative
iEEG	Intracranial electroencephalography
INCF	International Neuroinformatics Coordinating Facility
MEG	Magnetoencephalography
MIS	SPARC minimal information standard
MRI	Magnetic resonance imaging
NEMAR	NeuroElectroMagnetic data Archive
NIF	Neuroscience Information Framework
NIH	National Institutes of Health
NWB	Neurodata Without Borders
ODC-SCI	Open Data Commons for Spinal Cord Injury
ODC-TBI	Open Data Commons for Traumatic Brain Injury
PET	Positron emission tomography
SDS	SPARC dataset structure
SPARC	Stimulating Peripheral Activity to Relieve Conditions
SPECT	Single-photon emission computed tomography
URI	Uniform Resource Identifier

## Footnotes

^1^ http://cocomac.g-node.org/

^2^ https://www.incf.org/resources/sbps

^3^ https://bioportal.bioontology.org/

^4^ https://www.ebi.ac.uk/ols/index

^5^ https://bluebrainnexus.io/

^6^ https://globalbrainconsortium.org/
